# Transient Gerstmann syndrome as manifestation of stroke: Case report and brief literature review

**DOI:** 10.1590/1980-57642016dn11-020013

**Published:** 2017

**Authors:** Rafael Batista João, Raquel Mattos Filgueiras, Marcelo Lucci Mussi, João Eliezer Ferri de Barros

**Affiliations:** 1 Hospital Municipal Dr Jose de Carvalho Florence, Sao José dos Campos, SP, Brazil

**Keywords:** Gerstmann syndrome, disconnection syndrome, insular cortex, parietal lobe, frontal lobe, síndrome de Gerstmann, síndrome de desconexão, córtex insular, lobo parietal, lobo frontal

## Abstract

Gerstmann Syndrome (GS) is a rare neurological condition described as a group of cognitive changes corresponding to a tetrad of symptoms comprising agraphia, acalculia, right-left disorientation and finger agnosia. It is known that some specific brain lesions may lead to such findings, particularly when there is impairment of the angular gyrus and adjacent structures. In addition, the possibility of disconnection syndrome should be considered in some cases. The purpose of this article is to report a case of a young, cardiac patient, non-adherent to treatment, who presented with a stroke in which transient clinical symptoms were compatible with the tetrad of GS. The case report is followed by a discussion and brief review of the relevant literature.

## Abstract (pt)

A síndrome de Gerstmann (SG) é uma condição neurológica rara, caracterizada por um grupo de alterações cognitivas que correspondem a uma tétrade composta por agrafia, acalculia, desorientação direita-esquerda e agnosia para dedos. Sabe-se que certas lesões encefálicas específicas podem levar a tais achados, particularmente quando ocorre acometimento do giro angular e estruturas adjacentes. Além disso, a possibilidade de síndrome de desconexão deve ser considerada em alguns casos. O propósito deste artigo é relatar o caso de um paciente jovem cardiopata e não aderente ao tratamento que se apresentou com uma síndrome encéfalo-vascular associada a alterações clínicas transitórias compatíveis com a tétrade da SG. Este relato de caso é acompanhado de discussão e breve revisão de dados pertinentes da literatura.

## INTRODUCTION

Gerstmann's syndrome (GS) is a rare clinical condition that has been extensively studied by neuroscience researchers, particularly in the field of neuropsychology. Classically, this syndrome comprises a tetrad of symptoms: acalculia, agraphia, finger agnosia and right-left disorientation.^1^ The literature documents that lesions in the dominant hemisphere can result in these cognitive changes,^2^ which are occasionally associated with aphasic disorders.^3^ We report a case of transient GS in a young adult patient.

## CASE REPORT

A 43-year-old male right-handed patient, a student in use of illegal anabolic steroids with a 2-year history of mitral valve replacement (biological) and atrial fibrillation without regular follow-up, presented at the emergency room with complaints compatible with sudden onset of language impairments and right sensorimotor hemiparesis with faciobrachial predominance. On admission assessment, vital signs were stable and the blood glucose test ruled out hypo or hyperglycemia. The general clinical exam revealed irregular heartbeat, systolic murmur in the mitral region (3+/6+) radiating to the left midaxillary line, and no other abnormalities. On the neurological exam performed around 6 hours after ictus (a few minutes after patient arrival at hospital), the patient was alert, collaborative in performing the exam and spatially and temporally well-oriented. The strength exam revealed labial commissure deviation to the left and paresis of the proximal right upper limb (strength grade IV+). Results on coordination and sensory tests of all modalities were normal.

Neuropsychological assessment revealed aphasia characterized by low fluency (speech output of less than 10 words per minute); repetition inability; difficulty in naming simple objects and in spontaneous naming on word-list generation task (fewer than 12 animals per minute); absence of ideomotor apraxia for transitive and intransitive gestures; agraphia characterized by difficulty writing letters and words spontaneously, dictated or copied; acalculia for low complexity addition and subtraction sums; finger agnosia (inability to follow command to move specific finger of one hand upon touching of finger on opposite hand by examiner, in addition to difficulty recognizing fingers of own hand) and right-left disorientation, for example, when requested to point to right foot with left hand.

Cranial computed tomography (CT) exam on admission was normal while electrocardiogram disclosed irregular rhythm and absence of p wave, with no ischemic changes. It is important to note that no echocardiographer was available at the time to provide an emergency assessment.

**Figure 1:**
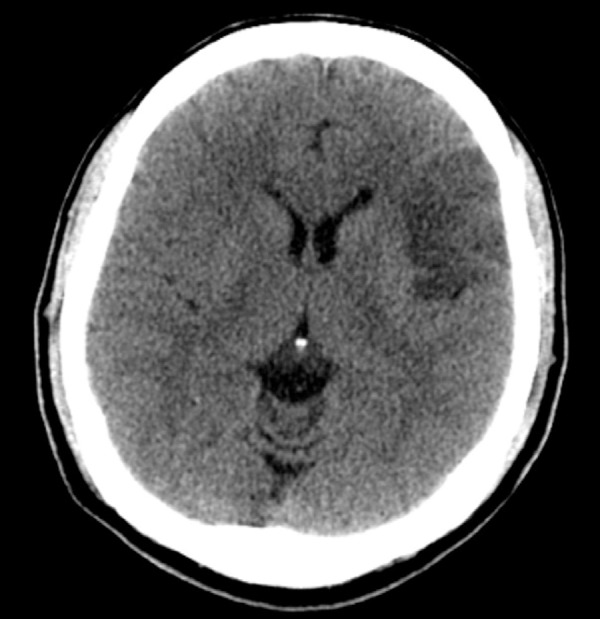
Cranial Computed Tomography in axial section showing hypodense area in the left inferior frontal gyrus topography.

**Figure 2:**
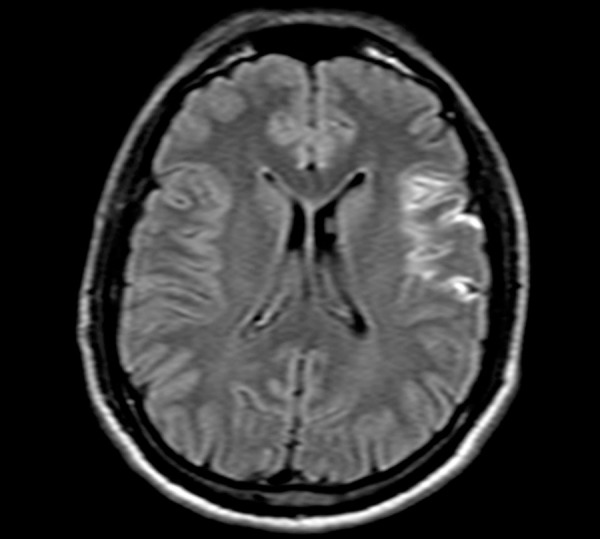
Brain Magnetic Resonance Imaging (FLAIR) in axial section showing hypersignal in the left anterior and posterior insular cortex topography, with sparing of the left angular and supramarginal gyri.

**Figure 3:**
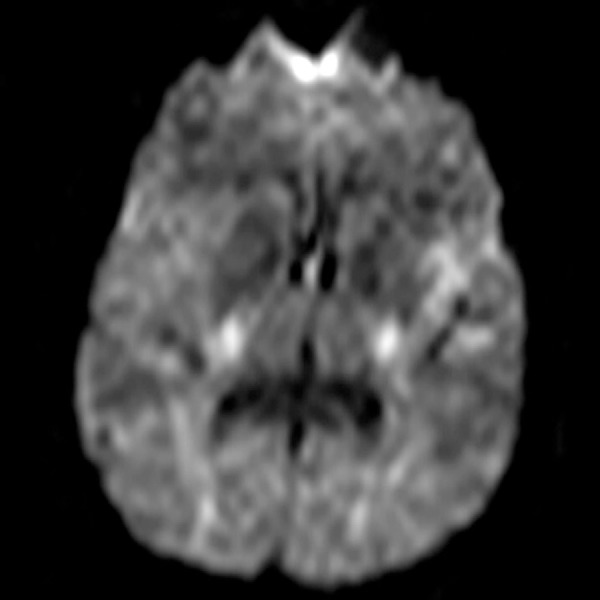
Brain Diffusion Magnetic Resonance Imaging in axial section showing restricted diffusion in the cortical region of the left insular topography.

At the time the patient was seen by the neurology team, he was considered ineligible for endovenous thrombolysis owing to the time interval of over four and a half hours between the onset of clinical symptoms and arrival at the emergency room. Submitted to clinical support, the patient attained a stable condition without complications. Approximately 30 hours after clinical symptoms onset, the patient had good general health status and no motor deficits. Another neuropsychological assessment revealed preserved comprehension, ability to produce complete simple and complex sentences, occasionally associated with difficulty in expression and in naming simple objects, and no abnormalities in writing letters, words or complete sentences, exhibiting preserved right-left orientation and finger gnosia in response to the same prompts given in the admissions exam. Cranial CT performed 72 hours post-ictus revealed a hypodense area in the left inferior frontal lobe (Figure 1). Brain Magnetic Resonance Imaging (MRI) performed during outpatient follow-up disclosed hypersignal and restricted diffusion in the left anterior and posterior insular cortex (Figures 2 and 3), with sparing of the topography corresponding to the angular and supramarginal gyri. During follow-up, the patient was referred for neuropsychological rehabilitation and to the outpatient clinic in use of warfarin as a secondary prophylactic.

## DISCUSSION

In 1924, Austrian neurologist Joseph Gerstmann described the case of a 53-year-old patient presenting with agraphia, acalculia, right-left disorientation and difficulty moving specific fingers when requested by the examiner, clinical findings related to cerebrovascular syndrome with left hemisphere involvement.^2^

Three years later, the same author reported two similar cases exhibiting constructional apraxia, anomia for colors and difficulty reading numbers but not words.^4^ However, it was in 1940, when Gerstmann's work was published in English, that the syndrome became known in the global academic milieu. In that same year, GS was characterized by the tetrad comprising: agraphia, acalculia, finger agnosia and right-left disorientation.^1^

In the decades that followed, a strong relationship between lesions in the dominant hemisphere and GS was confirmed, more specifically in the angular gyrus of the parietal lobe.^5-8^ Lesions in this gyrus are associated predominantly with the symptoms of agraphia and acalculia.^9^

In 1984, Morris et al. reported the case of a patient with transient findings compatible with GS after stimulation of the posterior perisylvian region, more specifically in the transition between the angular and supramarginal gyri.^10^ In 2003, Roux et al. found abnormalities involving writing, calculating and finger recognition using the brain mapping technique in the left angular gyrus of 6 right-handed patients submitted to tumor resection.^11^

Even in cases where the angular gyrus is spared, lesions immediately subcortical to this area, or involving the mesial occipital cortex or splenium of the corpus callosum, may also lead to agraphia and/or acalculia.^12^ In addition, in cases of GS with sparing of the classically involved areas, the hypothesis of disconnection syndrome involving lesions to association fibers joining different cortical and subcortical regions should also be considered.^13^

Seven years ago, Rusconi et al. revisited various aspects of GS, including their first description and the importance of the applicability of the concept of the disconnection syndrome in this context. The authors stated that certain recent studies employing the neurofunctional imaging technique, such as those performed using tractography, suggest that the group of clinical symptoms of GS perhaps should not be attributed exclusively to one specific neuronal group.^14^ This evidence again brought to the fore criticisms of Gerstmann made by some authors, such as Critchley, in the mid-1960s, when the localizing value of the syndrome was questioned.^15^

In view of these considerations, it is noteworthy to mention the insular cortex and its interaction with other areas. This structure has a reciprocal connection with the parietal operculum, anterior inferior parietal cortex, somatosensory cortex, retroinsular parietal region,^16^ orbitofrontal and pre-frontal cortex, frontal operculum and efferent pathways to the inferior frontal gyrus and Brodmann's areas 6 and 12.^17^ As is known, lesions located immediately deep to the insular cortex, more precisely in the extreme capsule, may cause disconnection of short association fibers between frontal and parietal operculae and lead to disruption of frontoparietal circuitry.^18,19^

In 2014, a case was reported of an elderly patient with dyscalculia, dysgraphia and right-left confusion (without finger agnosia) secondary to ischemic injury of the left posterior insula and temporal-parietal operculum, without lesions of the angular and supramarginal gyri.^19^

Recently, several articles have suggested a relationship between the frontal lobe and GS. In 2013, Heymi Lee et al. published two cases in which Brain MRI disclosed middle and inferior frontal gyri and basal ganglia impairment as a result of ischemic stroke with hemorrhagic transformation in the first case, and involvement of the inferior frontal gyrus, pars opercularis and triangularis secondary to the compression effect by glioma in the second case. Neurological exams in both patients revealed the four typical symptoms of GS.^20^

In 2016, Eun-Ju Lee et al. published two cases of patients that had clinical symptoms compatible with the GS tetrad, where ischemic lesion of the left medial frontal lobe was found on Brain MRI, with sparing of the angular and supramarginal gyri and adjacent structures. In these articles, the authors attributed the findings to the possibility of disconnection between the association fibers and suggested the importance of the cortical and subcortical regions of the left frontal lobe in the physiopathogenesis of GS, given there is a strong connection between these areas and the parietal lobe.^21^

Our review of the literature lead us to believe that the changes initially observed on the patient's neurological exam may have been attributed to mechanisms related to the disconnection syndrome secondary to ischemic injury of the territories of association fibers between frontal and parietal lobes, given that lesions were present in the inferior frontal gyrus and insular cortex of the dominant hemisphere.

Considering that the middle cerebral artery irrigates the insular cortex and segments of the frontal and parietal lobe, an alternative hypothesis for this case might be the possible occurrence of reperfusion in the penumbra area after clinical management.
